# Power Spectral Density and Functional Connectivity Changes due to a Sensorimotor Neurofeedback Training: A Preliminary Study

**DOI:** 10.1155/2019/7647204

**Published:** 2019-05-05

**Authors:** Juan L. Terrasa, Guzmán Alba, Ignacio Cifre, Beatriz Rey, Pedro Montoya, Miguel A. Muñoz

**Affiliations:** ^1^Research Institute on Health Sciences (IUNICS), University of Balearic Islands, 07122 Palma, Spain; ^2^Brain, Mind and Behavior Research Center, University of Granada (CIMCYC-UGR), 18011 Granada, Spain; ^3^University Ramon Llull, Blanquerna, FPCEE, 08022 Barcelona, Spain; ^4^Departamento de Ingeniería Gráfica, Universitat Politècnica de València, 46022 Valencia, Spain

## Abstract

Neurofeedback is a form of neuromodulation based on learning to modify some aspects of cortical activity. Sensorimotor rhythm (SMR) oscillation is one of the most used frequency bands in neurofeedback. Several studies have shown that subjects can learn to modulate SMR power to control output devices, but little is known about possible related changes in brain networks. The aim of this study was to investigate the enhanced performance and changes in EEG power spectral density at somatosensory cerebral areas due to a bidirectional modulation-based SMR neurofeedback training. Furthermore, we also analyzed the functional changes in somatosensory areas during resting state induced by the training as exploratory procedure. A six-session neurofeedback protocol based on learning to synchronize and desynchronize (modulate) the SMR was implemented. Moreover, half of the participants were enrolled in two functional magnetic resonance imaging resting-state sessions (before and after the training). At the end of the training, participants showed a successful performance enhancement, an increase in SMR power specific to somatosensory locations, and higher functional connectivity between areas associated with somatosensory activity in resting state. Our research increases the better understanding of the relation between EEG neuromodulation and functional changes and the use of SMR training in clinical practice.

## 1. Introduction

Several studies have shown that subjects can learn to self-regulate different parameters of the EEG activity (i.e., amplitude, frequency, and/or coherence of EEG signal) through neurofeedback training [1, 2]. Self-regulation of the sensorimotor rhythm (SMR, also known as central, Rolandic, or mu rhythm) is one of the most used neurofeedback training protocols [2, 3]. SMR refers to oscillations between 8 and 30 Hz recorded mostly over somatosensory areas [4]. Their amplitude decreases during real movement [5] or during motor imagination [6]. Specifically, a left/right hand motor imagery task shows a contralateral desynchronization (decreased amplitude) and ipsilateral synchronization (augmented amplitude) over somatosensory areas [7]. Several studies have shown that subjects can learn to self-modulate SMR amplitudes through motor imagery tasks to control output devices [8, 9]. Moreover, synchronization or desynchronization seems to be associated with different cognitive processes. Local synchronization has been associated with cortical idling and inhibition, whereas desynchronization has been related to active cognitive processes. Consequently, SMR desynchronization has been applied to recover motor function [10, 11], while SMR synchronization has been used to improve both attentional processes [12, 13] and working memory [14]. Furthermore, bidirectional modulation-based SMR neurofeedback training based on synchronization and desynchronization of the SMR during the same task has been also applied [15]. For example, in a recent sleep quality study, healthy subjects were able to successfully learn to modulate their SMR after 10 to 21 training sessions in C3 and C4 electrodes; besides, no effects on sleep were observed [16]. In the present study, a bidirectional SMR neurofeedback was assessed by using individually selected electrodes to train after a screening session.

Along with the large number of studies reporting success in training subjects to self-regulate EEG activity by using neurofeedback, some researches have also provided evidence of the training-related changes in brain networks. For example, a single EEG neurofeedback training session of alpha band desynchronization at Pz electrode has induced significant enhancements of functional connectivity of the anterior cingulate cortex within the salience network during an attentional task, as well as significant reductions of mind wandering [17]. In another study, a single neurofeedback session of alpha band desynchronization at Pz in posttraumatic stress disorder participants showed evidence of neuronal reconfiguration among areas such as insula, cingulate cortex, and amygdala, which are highly implicated in the disorder [18, 19]. However, little is known about changes in resting-state functional connectivity induced by SMR neurofeedback training. To the best of our knowledge, only one EEG neurofeedback study has addressed this issue. In an EEG motor imagery neurofeedback study in patients with stroke, participants had to imagine the movement of their stroke-affected limb towards a displayed goal. Resting-state fMRI analysis showed increased functional connectivity in motor cortices, the supplementary motor area, the visuospatial system, and the cerebellum due to the neurofeedback training. Moreover, these changes were associated with motor recovery [11]. These results are valuable to understanding how SMR neurofeedback translates into functional changes in the brain. However, the specifications of the EEG training were not clear enough to fully interpret this translation. Authors reported that feedback was provided to the participants when detecting motor imagery activity by using a subject-specific filter algorithm (bank common spatial pattern) over 27 EEG channels. This method is excellent for detecting cerebral patterns of motor imagery, but it seems to provide nonspecific information about on which channels and frequencies occur both the synchronization and desynchronization of the SMR. The present study is aimed at exploring the potential functional changes provoked by a bidirectional modulation-based SMR neurofeedback training, where participants had to synchronize and desynchronize the target frequencies in a specific location.

The main objective of the present study was to investigate the enhanced performance and changes in EEG power spectral density at somatosensory cerebral areas due to an SMR neurofeedback training. For this purpose, a protocol based on learning to synchronize and desynchronize (modulation) the SMR was designed. Our hypothesis was that participants trained with SMR neurofeedback protocol would show a better task performance and an enhanced SMR power modulation over somatosensory-related electrodes at the end of the training. Furthermore, the present study also analyzed the functional changes in somatosensory areas during resting state induced by a bidirectional modulation-based SMR neurofeedback training as exploratory procedure in half of the participants.

## 2. Materials and Methods

### 2.1. Participants

Thirty healthy female students (aged 19.1 ± 2.68) from the University of Granada (Spain) were enrolled in the study. All participants were right-handed and had normal or corrected-to-normal vision. Participants were not pregnant and were healthy without auditory deficits or neurological diseases. Participants were volunteers, and they received course credit for participation. The study was conducted in accordance with the Declaration of Helsinki (1991) and approved by the Ethics Committee of the Balearic Islands (Spain) (IB 2268/14 PI). Written informed consents were obtained from the participants after the experimental procedure explanation.

### 2.2. Procedure and Electroencephalography Data Acquisition

Upon arrival to the laboratory, participants were randomly assigned either to one SMR neurofeedback training (*n* = 10) or to one of the two control groups named SHAM and occipital (OCC) (*n* = 10 each group). As the neurofeedback protocol was based on visual cues, we expected occipital activity accompanying the SMR modulation. It is known that alpha rhythms originated in the occipital region are associated with visual attention processes [20], whereas alpha rhythms generated in the somatosensory cortex can be related to motor processes [21]. To differentiate between these effects, an occipital 12-15 Hz neurofeedback training protocol was assessed as control (OCC group) to ensure that the SMR modulation was reflecting somatosensory and not occipital activation. This frequency bin (12-15 Hz) was selected as the SMR frequency bin most used in EEG neurofeedback [2].

Participants were informed about the experiment, signed an informed consent, and completed the Spanish version of the Edinburgh Handedness Inventory (EHI) [22] and the Revised Movement Image Questionnaire (MIQ-R) [23]. Give that high anxiety levels can impair neurofeedback training [24, 25], all subjects completed the State-Trait Anxiety Inventory (STAI-S) [26] before each neurofeedback session. Finally, they rated their level of concentration and fatigue (1-7 scale) after each session.

All participants completed an initial EEG screening session followed by six EEG neurofeedback sessions. During EEG recording, subjects were comfortably seated in an armchair in a dimly lit room. The EEG signals were registered and amplified with a sampling rate of 512 Hz by an ANT amplifier (Neuro Asalab, ANT Neuro, Netherlands). A 50 Hz notch filter was applied. EEG was recorded from thirty Ag/AgCl electrodes placed according to the 10-20 International System referenced to the average, and the ground electrode was located at position AFz. Electrode impedance was kept lower than 10 kOhm. The feedback information was shown on a screen (2 meters in diagonal) situated at 2.5 meters in front of the participant. Participants had to mentally control the movement of a cursor (feedback) in order to hit a target which appeared in the left or right edge of the screen as many times as possible. The first and sixth sessions were considered as assessment sessions (PRE and POST, respectively) where all groups received real feedback about their performance (i.e., they actually controlled the cursor movement). In the rest of the sessions (second to fifth, considered as training), the SMR and OCC groups received real feedback of the target frequencies from the target locations as in the assessment sessions, whereas the SHAM group received random feedback (pseudorandom cursor movement elicited by a prerecorded session). In this case, the cursor was moved in 50% of the trials to the left and in 50% of the trials to the right and hitting the target only in half of the trials (the same number of trials for right and left targets). The same prerecorded session was applied to all participants in the SHAM group.

### 2.3. Functional MRI Data Acquisition

Changes in functional connectivity due to neurofeedback training were evaluated by two resting-state fMRI sessions. Half of participants (5 per group) were enrolled in two fMRI sessions conducted in different days than in the EEG sessions: the day before the screening session (rsPRE) and the day after the last EEG neurofeedback session (rsPOST). During these sessions, participants remained with their eyes closed for 8 minutes while fMRI was recorded. Data were acquired using a 3.0-Tesla scanner (SIEMENS MAGNETOM TrioTim syngo MR) located at the Mind, Brain and Behavior Research Center of the University of Granada (CIMCYC-UGR). Echo-planar sequence (EPI) functional images were acquired for 8 minutes of eyes-closed resting for each subject before (rsPRE) and after (rsPOST) the neurofeedback sessions (total volumes = 240, 32 slices per volume interleaved, TR = 2.0 s, ET = 23 ms, flip angle = 80°, acquisition matrix = 68 × 68, FOV = 224 mm, slice thickness = 3.5 mm, and no gap). MPRAGE sequence T1 anatomical images were also acquired for each subject to perform coregister and nuisance preanalyses (176 slices, TR = 2.52 ms, flip angle = 9°, FOV = 256 mm, and slice thickness = 1 mm).

### 2.4. Screening Session

**Figure 1:**
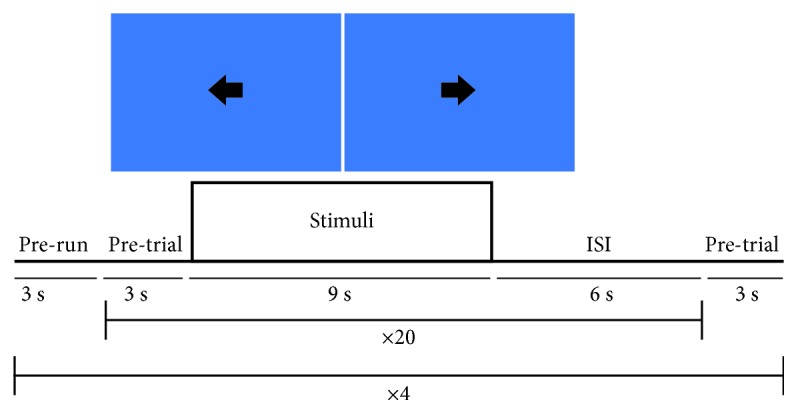
Experimental design of the screening session.

The screening session (Figure 1) was planned in order to extract the individual EEG features (i.e., electrode location and target frequency) to be used in the following neurofeedback sessions. As SMR spatial patterns and exact frequencies were different across individuals, we decided to use individually determined features to optimize the learning to self-modulate SMR. Before the screening session, participants were trained to pay attention to sensations of opening and closing hands by asking them to repeatedly squeeze a soft ball. Then, participants had to imagine the same hand movements. The screening session began only when participants were able to successfully imagine opening and closing the hands. The screening task was programmed using the stimulus presentation module of the BCI2000 platform [27] and consisted of 4 runs, with 20 trials each (10 trials for left hand and 10 trials for right hand motor imagery) presented in pseudorandomized order. Each trial started with the presentation of an arrow (stimulus) pointing to the left (left hand) or to the right (right hand) specifying the motor imagery task to perform for 9 seconds until the arrow disappeared, followed by a 6-second interstimulus interval. The participants had to imagine the kinesthetic experience [28] of opening and closing left and right hands while EEG was recorded.

In order to detect the EEG features with the largest differences between left and right conditions, an offline analysis was performed. EEG data were divided into 9-second epochs depending on the two conditions (right vs. left), and a spectral analysis was performed by means of maximum entropy method with a resolution of 2 Hz for all frequencies between 0 and 70 Hz. The coefficient of determination *r*^2^ was calculated over spectral power to determine differences in the values of each feature in the two conditions. Finally, *r*^2^ values were compiled in a channel-frequency matrix and head topography. During all the following neurofeedback sessions, each participant of the SMR and SHAM groups trained a specific hemisphere and localization according to which electrodes showed a greater *r*^2^ value, i.e., the greater difference between right and left motor imagery in the screening session. Specifically for SMR and SHAM groups, the three electrodes placed in the fronto-central and/or centro-parietal areas of one hemisphere at a 3 Hz frequency bin within the sensorimotor rhythm (10-23 Hz) with larger *r*^2^ values were individually selected (Table 1). The range of possible frequencies covers upper alpha and beta given the great within-group variability of ranges obtained in the screening session. SMR subjects received real feedback information during all the sessions while SHAM participants received a random feedback in the training sessions (second to fifth neurofeedback sessions). Finally, for the OCC group three electrodes placed at O1, Oz, and O2 were selected and they received real feedback of their 12-15 Hz activity during all the sessions regardless the results of their screening session. As a control group, the frequency bin for OCC was selected because it is one of the most used in previous SMR neurofeedback literature [2].

### 2.5. Neurofeedback Sessions

**Figure 2:**
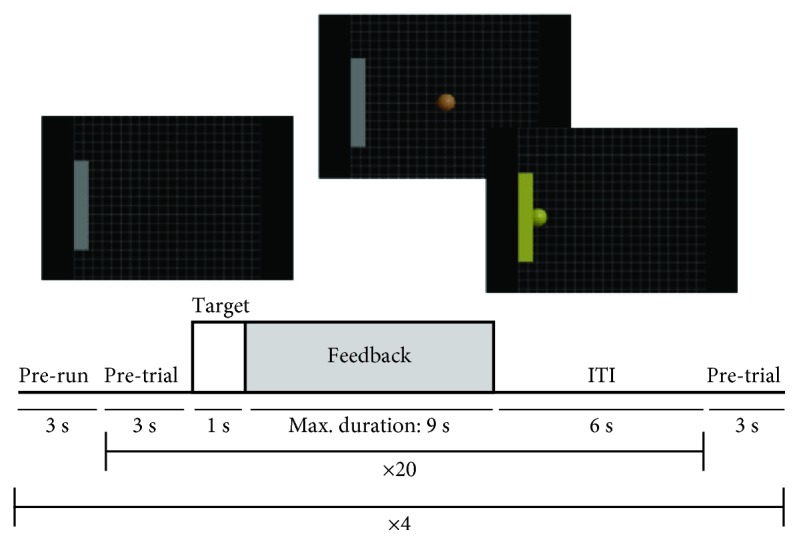
Experimental design of each of the six neurofeedback sessions.

Participants performed six neurofeedback sessions. Some of the subjects had two sessions per week (three weeks total) and some three per week (two weeks total). The neurofeedback task (Figure 2) was performed by using the Cursor Task module of the BCI2000 platform. Each trial started with the presentation of a target goal (a grey vertical rectangle) located at the left or the right edge of the screen. One second later, a brown ball (cursor) appeared in the middle of the screen. The subjects had to control this cursor over the horizontal axis and had a maximum of 9 seconds to move the cursor and impact the target. If they succeeded, the target and the cursor changed the color to yellow for one second (reward) and then disappeared. Otherwise, the cursor simply disappears. Participants received no instruction besides that they had to learn to “mentally” control the cursor (move it to the right or to the left depending on the target position) without any kind of body or facial movement, and they had to hit the target as many times as possible. However, we expected participants to be able to use similar strategies than in the screening session (motor imagery) at some point of their training. Ideally, the subjects had to synchronize or desynchronize the power spectrum of their target features (Table 1) to move the cursor in one direction or another. Each session consisted of 4 runs and 20 trials for each run with a 6-second intertrial interval.

During each neurofeedback training session, spectral power is calculated by BCI2000 every 0.5 seconds of input data by means of the maximum entropy method (autoregressive model order = 16) with 3 Hz bin resolution. These signal features were translated into output control signal using a linear equation selecting the spectral power of the three target electrodes into the target 3 Hz frequency bin. Finally, data were normalized to make the output control signal with mean of zero and variance of the unit by using a 6-second buffer of the feedback phase per condition (left or right target) to estimate the offset and gain values. Thus, there were two independent buffers, one for synchronization trials and one for desynchronization. For example, one buffer stored data only during synchronization feedback trials overwriting its oldest data once the buffer was filled (6 seconds). The offset and gain values for normalization were updated during the intertrial interval (never within the feedback trials). The participants were not told when they had to synchronize or desynchronize the EEG rhythms, but they were instructed where to move the cursor (left or right). For example, a participant of the SMR group who had to modulate SMR spectral power at three electrodes located over the left hemisphere had to generate a SMR synchronization to move the cursor to the left and a SMR desynchronization to move the cursor to the right. A second participant with the trained electrodes located over the right hemisphere had to synchronize the SMR to move the cursor to the right, but to desynchronize the SMR to move the cursor to the left. Regardless of synchronization or desynchronization, both participants had to ideally imagine right-hand movements to move the cursor to the right and to ideally imagine left-hand movements to move the cursor to the left. In addition, the speed of the cursor movement was greater the greater was the change in power spectra.

### 2.6. Demographic and Psychological Data Analysis

One-way analysis of variance (ANOVAs) was used to examine possible differences between groups (SMR, OCC, or SHAM) in age and in EHI and MIQ-R scales. The differences in STAI-S, concentration, and fatigue scores were tested by using a 3 × 6 ANOVA with group as a between-group factor and session (six sessions) as a repeated-measures factor on each dependent variable.

### 2.7. Task Performance Data Analysis

Task performance success (percentage of hits: cursor impacts on target within the time limits) was tested by using a 3 × 2 ANOVA with group as a between-group factor and evaluation session (PRE and POST) as a repeated-measures factor on each dependent variable. In order to further analyze the differences across all the training, an additional ANOVA was computed by using the within-subject factor session (6 sessions).

### 2.8. Electroencephalography Data Preparation and Analysis

Data preparation and statistical analysis were carried out using Matlab R2015b. In a first step of analysis, all EEG data were bandpass-filtered using a finite impulse response (FIR) filter with 1 Hz as the lower-edge frequency and 30 Hz as the higher-edge frequency. Furthermore, the Gratton & Coles algorithm was applied for ocular correction. Data were divided into 9-second epochs separately for those trials in which subjects were synchronizing (increasing amplitudes at target frequency) and desynchronizing (decreasing amplitudes at target frequency). Then, power spectral density (PSD) was calculated for the 1-30 Hz interval at all channels at 1 Hz resolution by using Welch's overlapped segment averaging estimator. The PSD data used in further statistical analyses were computed for each participant using the mean PSD of the three specific electrodes and within the 3 Hz frequency bin trained during the neurofeedback sessions. In order to minimize individual differences and to ensure comparability across participants [29], relative PSD (rPSD) values were estimated as the ratio of the computed PSD to total power across the 1-30 Hz range for each participant. Finally, the data were pooled within each group (SMR, OCC, and SHAM) and by the task (synchronization or desynchronization), regardless of which electrodes (located at right or left hemisphere) and frequencies that were individually trained.

In order to compare SMR, OCC, and SHAM groups in their learning to synchronize or desynchronize as a result of neurofeedback training in each localization, 3 × 2 × 2 ANOVAs were performed with group as the between-group factor and evaluation session (PRE, POST) and location (somatosensory or occipital electrodes) as repeated-measures factors. Regarding location factor, the somatosensory data for SMR and SHAM groups were selected from the trained electrodes in the evaluation sessions, while for OCC group were selected from the electrodes with larger *r*^2^ obtained in the screening session (nontrained electrodes). The occipital data were obtained from Oz O1 and O2 for all groups, regardless there were trained electrodes (OCC group) or not (SMR and SHAM groups).

In addition, changes on SMR at EEG electrodes located over somatosensory and motor cortices were tested by using 3 × 2 × 2 ANOVAs, with training group (SMR, OCC, and SHAM) as a between-group factor and evaluation session (PRE vs. POST) and modulation task (synchronization vs. desynchronization) as repeated-measures factors. Similar analyses were also carried out by using the occipital electrode locations. As mentioned earlier, the data from each location (somatosensory or occipital areas) were grouped regardless of the hemisphere they were obtained from.

In all the analyses involving repeated measures, the Greenhouse–Geisser epsilon correction was applied to control for violation of the sphericity assumption. Results are reported with the original degrees of freedom, the corrected *p* values, and the partial eta squared. When significant effects were found, post hoc analyses were performed using Bonferroni correction. The level of significance was set at 0.05. All analyses were performed using IBM SPSS Statistics v21.

### 2.9. Functional MRI Data Preparation and Analysis

Preprocessing of fMRI data was performed with Data Processing Assistant for Resting-State fMRI (DPARSF 2.4, http://www.restfmri.net) [30], based on the Statistical Parametric Mapping (SPM12) program (http://www.fil.ion.ucl.ac.uk/spm) and the Resting-State fMRI Data Analysis Toolkit (REST 1.8, http://www.restfmri.net) [31]. In order to stabilize the signal, the first 5 functional volumes were erased. Slice timing correction and head motion correction were performed (no subject presented more than 2 mm or 2° motion). Data were realigned to correct small movements and normalized to an MNI space (3 × 3 × 3 mm voxels) using the anatomical segmentation of T1. The linear trend of the time courses was removed, and a temporal bandpass filter was used (0.01-0.1 Hz). Data were smoothed with a 4 × 4 × 4 mm FWHM Gaussian kernel.

To analyze functional connectivity, a region of interest (ROI) to ROI correlation analysis was performed for each subject. The following ROIs were selected for all groups: bilateral postcentral gyri, precentral gyri, and supplementary motor area as somatosensory and motor-related areas (six ROIs in total) and bilateral calcarine, cuneus, and lingual as visual-related areas (six ROIs in total). The averaged time course obtained from each of the twelve ROIs was individually correlated with the rest of the automated anatomical labelling (AAL) cerebral ROI mean signal (*n* = 90, ROIs from the cerebellum and vermis were not selected). The significance threshold was set to *p* < 0.001 to avoid multiple-comparison error.

## 3. Results

### 3.1. Demographic and Psychological Data

ANOVAs revealed no significant group differences on age (*F* [2, 29] = 1.478, *p* = 0.246, *ηp*^2^ = 0.099), EHI (*F* [2, 29] = 0.834, *p* = 0.445, *ηp*^2^ = 0.058), MIQ-R (*F* [2, 29] = 0.290, *p* = 0.750, *ηp*^2^ = 0.021), STAI (*F* [2, 27] = 1.209, *p* = 0.314, *ηp*^2^ = 0.082), concentration (*F* [2, 27] = 1.868, *p* = 0.174, *ηp*^2^ = 0.122), or fatigue (*F* [2, 27] = 0.287, *p* = 0.753, *ηp*^2^ = 0.021) (Table 2). Neither were there any group differences on age (*F* [2, 14] = 1.018, *p* = 0.390, *ηp*^2^ = 0.145), EHI (*F* [2, 14] = 1.715, *p* = 0.221, *ηp*^2^ = 0.222), MIQ-R (*F* [2, 14] = 0.767, *p* = 0.486, *ηp*^2^ = 0.113), STAI (*F* [2, 12] = 0.988, *p* = 0.401, *ηp*^2^ = 0.141), concentration (*F* [2, 12] = 3.810, *p* = 0.052, *ηp*^2^ = 0.388), or fatigue (*F* [2, 12] = 0.190, *p* = 0.829, *ηp*^2^ = 0.031) when subjects participating in the fMRI assessment sessions were separately analyzed (Table 3).

### 3.2. Task Performance Success

**Figure 3:**
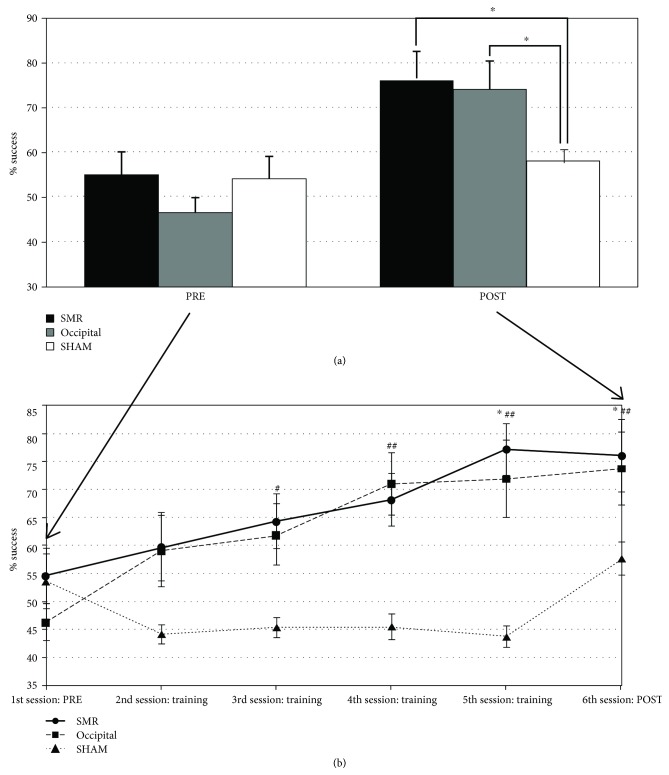
Percentage of success (hit the target). (a) Percentage of success of each group at PRE and POST sessions. ^∗^*p* < 0.05. (b) Percentage of success of each group across sessions. ^∗^Significant differences from PRE in the SMR group. ^#^Significant differences from PRE in the occipital group. ^∗#^*p* < 0.05 and ^##^*p* < 0.01.

Figure 3 shows the success level (percentage of hits) for each group in the PRE and POST sessions. The ANOVA revealed a significant effects of sessions (*F* [1, 27] = 25.546, *p* = 0.000, *ηp*^2^ = 0.486) and sessions × group (*F* [2, 27] = 4.217, *p* = 0.025, *ηp*^2^ = 0.238). Post hoc mean comparisons of the interaction effect revealed no significant differences between groups in percentage of hits at the PRE session, whereas significant group differences appeared between SMR (75.75%±18.68) and OCC (74.88%±19.90) participants compared to SHAM (57.62%±9.29) at the POST session (*p* = 0.022 and *p* = 0.028, respectively).

Figure 3 also displays the percentage of hits for each group through the six neurofeedback sessions. The ANOVA revealed a significant main group effect (*F* [2, 27] = 7.764, *p* = 0.002, *ηp*^2^ = 0.365), as well as significant effects of sessions (*F* [5,135] = 10.668, *p* = 0.000, *ηp*^2^ = 0.283) and sessions × group (*F* [10,135] = 3.804, *p* = 0.003, *ηp*^2^ = 0.220). Post hoc analyses of the main group effect comparisons yielded significant differences between SMR (mean of the six sessions = 66.62%±13.69) and SHAM groups (48.31%±4.78) (*p* = 0.003), as well as between OCC (63.96%±13.03) and SHAM (*p* = 0.012), whereas no significant differences were found between SMR and OCC groups. Furthermore, post hoc mean comparisons of the interaction effect revealed that SMR participants enhanced their performance in the 5th (*p* = 0.019) and 6th (*p* = 0.028) sessions compared with the 1st session (PRE). In addition, the OCC group showed a significant enhancement of their performance across the sessions starting already at the 3rd session (*p* = 0.050, *p* = 0.006, *p* = 0.006, and *p* = 0.001, respectively).

### 3.3. Electroencephalography Changes

**Figure 4:**
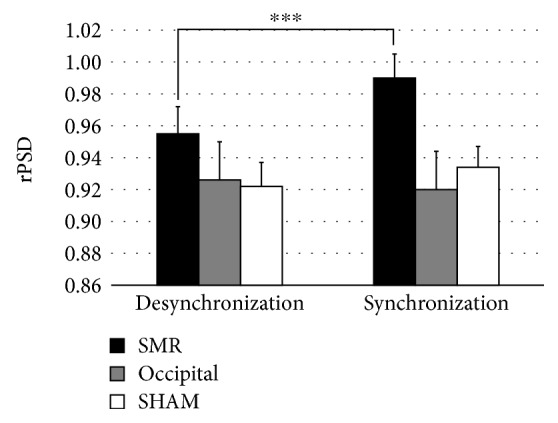
Relative spectral density (rPSD) during synchronization and desynchronization over somatosensory-related electrodes by each group in PRE and POST sessions. ^∗∗∗^*p* < 0.001.

Figure 4 displays the relative power spectral density (rPSD) over somatosensory-related electrodes (Table 1) during synchronization and desynchronization at the PRE and POST sessions for each training group. Taking into account the somatosensory-related electrodes, a 3 × 2 × 2 ANOVA on rPSD showed significant effects of modulation task (*F* [1, 27] = 7.819, *p* = 0.009, *ηp*^2^ = 0.225) and modulation task × group (*F* [2, 27] = 5.642, *p* = 0.009, *ηp*^2^ = 0.295). Post hoc comparisons revealed significant differences between rPSD synchronization (0.990 ± 0.076) and desynchronization (0.955 ± 0.079) of the target frequency (*p* = 0.000) only in the SMR group. This effect was observed in both sessions (PRE and POST). No significant differences due to sessions were found. Finally, the ANOVA of the rPSD over occipital electrodes yielded no significant effects.

**Figure 5:**
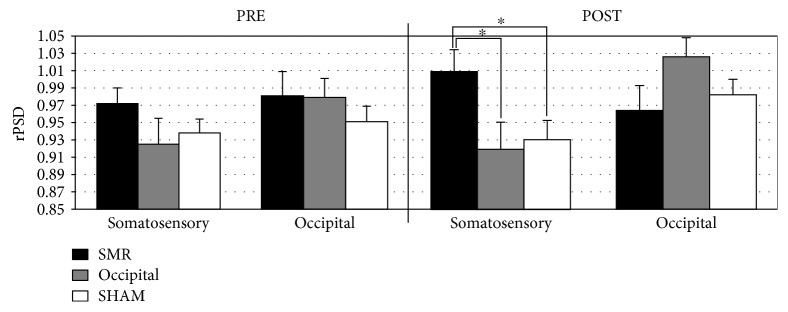
Relative power spectral density (rPSD) during synchronization in PRE and POST sessions over somatosensory-related electrodes and occipital electrodes. ^∗^*p* < 0.05.

In order to further analyze the effects of neurofeedback training, two ANOVAs with the factors evaluation session (PRE vs. POST), location (somatosensory related vs. occipital electrodes), and training group were separately performed on rPSD during synchronization and desynchronization. During synchronization, a significant effect of evaluation session × location × group was observed (*F* [2, 27] = 3.803, *p* = 0.035, *ηp*^2^ = 0.220). Post hoc comparisons revealed significant differences on somatosensory-related electrodes between SMR (1.008 ± 0.085) and OCC participants (0.919 ± 0.085), as well as between SMR and SHAM (0.930 ± 0.079) (*p* = 0.037 and *p* = 0.041, respectively) in the POST session (Figure 5). No significant differences in post hoc comparisons were found on occipital electrodes. Finally, during desynchronization, no significant differences were yielded on rPSD.

### 3.4. Connectivity Changes

Table 4 displays the rsPRE to rsPOST connectivity changes between ROIs. The SMR group showed significant changes from rsPRE to rsPOST on functional connectivity of precentral, postcentral, and supplementary motor area ROIs. After the training, higher functional connectivity was observed between postcentral gyrus and ROIs from the middle frontal gyrus and lateral inferior occipital and precentral gyrus (all left). Similarly, there was a higher functional connectivity between the precentral gyrus and ROIs from the lateral inferior occipital (left) and inferior frontal gyrus, middle frontal gyrus, and precentral gyrus (all right). Furthermore, the supplementary motor area displayed also a higher connectivity with the precentral gyrus (left) (all *p* < 0.001). Finally, no significant changes were observed in the occipital ROIs on functional connectivity. In the OCC and SHAM groups, no significant changes on functional connectivity from rsPRE to rsPOST were observed for any somatosensory or visual ROI.

## 4. Discussion

The aim of the present study was to examine the changes on EEG power spectral density at somatosensory and motor cerebral areas elicited by an SMR neurofeedback protocol. In order to test that neurofeedback training led to a better self-regulation of the sensorimotor rhythm (SMR group), a control group was trained with noncontingent feedback (SHAM group). Moreover, to check the specificity of the neurofeedback-related changes over somatosensory brain areas, a third group of participants were trained to self-regulate brain oscillations in the range 12-15 Hz at occipital brain locations (OCC group). Our findings suggest that the SMR group succeeded to self-regulate sensorimotor rhythm, as they showed a significant improvement on accuracy through sessions and these improvements were evidenced by an increased rPSD at somatosensory-related electrodes. We also observed that behavioral performance was improved in OCC participants across the sessions and that they learned to self-regulate brain oscillations over occipital electrode locations. A second purpose of this study was to explore the impact of EEG neurofeedback training over fMRI connectivity. Although it was a preliminary study and results should be taken with caution due to the small sample size, an increase in fMRI connectivity of somatosensory and motor-related areas in resting state was observed in the SMR group.

SMR and OCC groups showed significant increased accuracy through sessions. In contrast, no accuracy changes were found in the SHAM group through sessions. Our task performance results were consistent with previous studies which have shown that participants can learn to modulate sensorimotor rhythm [8]. Moreover, these studies show that participants reach similar scores to our study (about 75%) of task performance at the end of training [9]. These results confirm previous research indicating that visual feedback clearly modulates EEG rhythms when the feedback provides continuous outcomes of mental actions [32, 33]. It is important to highlight that it has been reported that about 20% of individuals cannot modulate their cerebral activity [34]. Several approaches have been proposed to enhance this modulation success in motor imagery tasks, e.g., by using vibratory stimulation producing kinesthesia experiences which are later mentally simulated during the motor imagery task [35, 36]. In the present study, the use of the screening session for the selection of individual electrodes used in the neurofeedback training was enough to obtain the expected success levels.

No differences between groups were found neither in handedness score (all the participants were right-handed) nor in their ability of imagining the movement without physically performing the movement (MIQ-R scores). Thus, all groups had the same kinesthetic imagination capacity. Furthermore, no differences in anxiety (measured with STAI-S) were found between groups at any session. Consistent with previous literature [37], anxiety was not related with the level of task success. Finally, no differences in concentration and fatigue scores were found between groups. Hence, none of these factors played a role in the neurofeedback training-increased accuracy.

The level of success on the task indicates that it was possible to learn to modulate sensorimotor rhythm in a unique location. The SMR group was able to successfully synchronize the EEG to move the cursor in one direction, as well as to desynchronize it to move the cursor in the opposite direction. The SMR training group was the only one with significant differences between SMR synchronization and desynchronization during the two evaluation sessions. The neural correlate of this successful training in the SMR group was an augmentation of sensorimotor rhythm in target electrodes when comparing PRE and POST evaluation sessions. Furthermore, sensorimotor rhythm at somatosensory-related electrodes was significantly higher in SMR than in both OCC and SHAM groups. This specific target-trained band change is an important issue not always reported in previous studies, as many authors observed positive behavioral results despite that no EEG-trained band changes were reported [38]. In addition, the present study was able to control other potential processes involved in the neurofeedback task, such as visual attention, through the OCC training group. Alpha rhythms originated in the occipital region are associated with visual attention processes [20] while alpha rhythms generated by the somatosensory cortex are related to motor processes [21]. The fact that the SMR group showed sensorimotor rhythm changes in contrast with the OCC group ensures that these changes reflect somatosensory activation and not visual activation. Hence, the present research seems to indicate that the reported EEG changes were specific in terms of frequency and localization with the somatosensory process.

It is well known that a left/right-hand motor imagery task elicits a contralateral desynchronization and ipsilateral synchronization of SMR over somatosensory areas [7]. In the present study, however, differences on sensorimotor rhythm power among the SMR group and the other groups were observed only during synchronization of the target frequency band. The absence of differences during desynchronization is difficult to explain. In this sense, it has been reported that some participants could manage the desynchronization of sensorimotor rhythm, but they are not able to sustain this attenuation long enough until the end of the feedback trial [8]. Thus, it could be that our participants were not able to sustain the SMR desynchronization long enough. Another possible explanation is based on the type of mental strategy used by the participants during the neurofeedback training. Predefined specific mental strategies have been successfully used in neurofeedback [39]. However, it has been reported that subjects who did not use specific mental strategies were those who showed linear improvements in performance during the neurofeedback training when compared with those using more specific mental strategies [40]. In the present study, although the participants were not told to use a specific mental strategy, we expected them to assume strategies like those used in the screening session (motor imagery) at some point in their training. Thus, it is possible that these “nonspecific” strategies based on a specific motor imagery task were only sufficient to differentiate SMR power changes between groups during synchronization but not during desynchronization.

Previous studies have suggested that neurofeedback training could induce long-term changes on brain activation and functional connectivity in several diseases [18, 19, 41]. Our findings indicate that the SMR neurofeedback training could cause long-term changes in functional connectivity and that brain networks could be shaped by experience-driven modulation as the SMR group yielded significant changes between rsPRE and rsPOST sessions. Higher functional connectivity was found in rsPOST when compared to rsPRE between the three ROIs associated with somatosensory activity (postcentral gyrus, precentral gyrus, and supplementary motor area) with several ROIs related to working memory (middle frontal gyrus), task decision-making (inferior prefrontal gyrus), and visual processing (lateral inferior occipital) [42, 43]. These results are in the line with a previous research that evaluated changes in resting state caused by SMR neurofeedback. For instance, increased functional connectivity in motor cortices, the supplementary motor area, the visuospatial system, and the cerebellum was found in patients with stroke after a motor imagery neurofeedback training [11]. However, the translation between motor imagery electrophysiological features and the reported functional changes were not clear enough. Our research highlights the functional changes elicited by bidirectional modulation-based SMR neurofeedback training. In this sense, the successful synchronization and desynchronization of SMR were mirrored in higher functional connectivity of somatosensory-related areas during resting state, regardless of the trained hemisphere. However, the fact that participants trained the SMR modulation in different hemispheres should be considered as a limitation of our fMRI analyses.

Finally, it is important to note that participants in the OCC group performed so successfully as did participants in the SMR group. Nevertheless, behavioral improvements in this group appeared with a nonsignificant trend to increased power in occipital brain areas during POST as compared to PRE. In addition, no significant changes from rsPRE to rsPOST were observed in functional connectivities of visual ROIs. It seems that mental strategies used by the OCC group participants to achieve a success level of task control were very variable and unspecific. It may be argued that, although participants learned to modulate their occipital activity during the neurofeedback sessions, these unspecific strategies were not powerful enough to observe functional changes in resting state, as compared to sensorimotor training.

The results of the present study should be taken with caution due to the following limitations. First, although the selection of individual electrodes for neurofeedback training was a good way to personalize neurofeedback, it would have been convenient to have a larger sample of subjects to analyze the possible effects of the different locations of the electrodes. Second, the fact that all participants were only women could have biased the results and, therefore, additional studies should include male participants to assess possible gender effects. Most importantly, the sample size for the resting-state fMRI analyzes was small and makes the functional findings only preliminary. Additional investigations with a larger sample size should be conducted to assess whether the effects of neurofeedback training of the SMR on the functional connectivity of the somatosensory-related regions are robust enough.

## 5. Conclusions

In summary, the present study revealed that neurofeedback training based on self-regulation of the SMR rhythm led to better performance and better discrimination between synchronization and desynchronization of brain rhythms for the group specifically trained in somatosensory areas compared to the group trained in the occipital region. Furthermore, the SMR neurofeedback could lead to functional changes during resting state as higher functional connectivity was found in areas associated with somatosensory activity. Finally, our research contributes to a better understanding of the relationship between EEG neuromodulation and functional changes associated with sensorimotor rhythm training.

## Figures and Tables

**Table 1:** Electrodes and frequencies trained by each participant of all groups and the mean coefficient of determination (*r*^2^) values of these features obtained in the screening session.

	Electrodes	Frequencies (Hz)	Mean *r*^2^
SMR group			
#1	C3 CP5 CP1	14-17	0.1378
#2	FC1 C3 CP1	20-23	0.1394
#3	C3 CP5 CP1	10-13	0.5856
#4	C4 CP2 CP6	20-23	0.1792
#5	C4 CP2 CP6	17-20	0.2240
#6	FC2 FC6 C4	17-20	0.1718
#7	C3 CP5 CP1	17-20	0.1673
#8	C4 CP2 CP6	20-23	0.0928
#9	C4 CP2 CP6	20-23	0.2347
#10	C4 CP2 CP6	20-23	0.1859

SHAM group			
#11	C3 CP5 CP1	20-23	0.0643
#12	C3 CP5 CP1	15-18	0.2394
#13	C4 CP2 CP6	20-23	0.1769
#14	C4 CP2 CP6	15-18	0.4980
#15	C3 CP5 CP1	20-23	0.0493
#16	C3 CP5 CP1	10-13	0.1024
#17	FC2 FC6 C4	12-15	0.2008
#18	C4 CP2 CP6	20-23	0.1053
#19	C4 CP2 CP6	10-13	0.0303
#20	C3 CP5 CP1	15-18	0.1008

OCC group			
#21-30	O1 Oz O2	12-15	—

**Table 2:** Mean and standard deviation of demographic and psychological data of all the participants by group.

	Session	SMR (*n* = 10)	OCC (*n* = 10)	SHAM (*n* = 10)
Age (mean, SD)		19.3 (3.466)	20 (2.981)	18 (0)

EHI (mean, SD)		22.9 (7.445)	20.6 (7.905)	19.1 (3.725)

MIQ-R (mean, SD)		18 (3.916)	18.6 (3.777)	19.4 (4.624)

STAI (mean, SD)	S1	17.9 (6.262)	19.8 (4.590)	16.6 (9.559)
	S2	18.6 (10.676)	22.5 (6.687)	14.1 (5.990)
	S3	19.8 (12.255)	19.6 (7.412)	16.1 (9.374)
	S4	18.3 (10.541)	20.1 (6.951)	20 (10.760)
	S5	18.7 (11.235)	25.1 (7.965)	17 (8.233)
	S6	17.9 (9.643)	18.7 (9.978)	15.2 (5.992)

Concentration (mean, SD) 1-7 scale (1: any, 7: maximal)	S1	4.9 (0.994)	4.8 (0.919)	5.3 (0.823)
	S2	4.5 (1.354)	4.6 (1.174)	4.7 (1.252)
	S3	5 (1.504)	4.1 (1.197)	4.8 (1.229)
	S4	5.4 (0.843)	4.9 (0.568)	5 (1.247)
	S5	5.2 (1.033)	4.6 (0.966)	5.5 (0.972)
	S6	5.3 (1.059)	4.5 (1.509)	6 (1.155)

Fatigue (mean, SD) 1-7 scale (1: any, 7: maximal)	S1	3.2 (1.751)	2.9 (1.595)	3.3 (1.494)
	S2	4 (1.414)	2.6 (1.350)	3.2 (1.549)
	S3	3.6 (1.776)	3.4 (1.430)	3.5 (1.434)
	S4	3.5 (1.581)	3 (1.633)	3.7 (1.059)
	S5	3 (1.491)	2.9 (1.792)	3.5 (1.269)
	S6	3 (1.633)	3.4 (1.578)	1.9 (0.994)

**Table 3:** Mean and standard deviation of demographic and psychological data of the fMRI performers.

	SMR (*n* = 5)	OCC (*n* = 5)	SHAM (*n* = 5)
Age (mean, SD)	18.4 (0.894)	19.2 (2.168)	18 (0)
EHI (mean, SD)	23.4 (7.797)	17.4 (2.510)	18.4 (4.827)
MIQ-R (mean, SD)	19.4 (4.278)	17.2 (4.324)	20 (2.345)
STAI-S (mean, SD)	17 (10.163)	19.9 (2.37)	13.5 (6.975)
Concentration (mean, SD)	5.5 (0.321)	4.6 (0.584)	5.6 (0.887)
Fatigue (mean, SD)	2.7 (0.893)	2.6 (0.572)	2.9 (0.847)

**Table 4:** Region of interest (ROI) to ROI correlation analysis of the SMR group.

SMR group	Cluster size	Cluster *p*	Cluster max *z*	*x*	*y*	*z*
Area						
PRE < POST ROI = *postcentral gyrus L*						
Middle frontal gyrus L	19	<0.001	3.73	-39	15	51
Lateral inferior occipital L	16	<0.001	3.69	-30	-90	3
Precentral gyrus L	10	<0.001	3.68	-48	-3	33
PRE < POST ROI = *postcentral gyrus R*						
—	—	n.s.	—	—	—	—
PRE < POST ROI = *precentral gyrus L*						
Lateral inferior occipital R	10	<0.001	3.75	57	-72	0
PRE < POST ROI = *precentral gyrus R*						
Inferior frontal gyrus L	27	<0.001	3.89	-48	42	-3
Middle frontal gyrus L	11	<0.001	3.88	-36	15	51
Precentral gyrus L	10	<0.001	3.83	-45	-3	33
PRE < POST ROI = *supplementary motor area L*						
Precentral gyrus L	12	<0.001	4.03	-51	-6	30
PRE < POST ROI = *supplementary motor area R*						
—	—	n.s.	—	—	—	—

## Data Availability

The data used to support the findings of this study are available from the corresponding author upon request.
